# Correlation and clinical significance of placental tissue selectin (E), angiotensin II and its receptors, and oxidized lipid levels in patients with preeclampsia

**DOI:** 10.5937/jomb0-51303

**Published:** 2025-01-24

**Authors:** Gao Yan, Wu Guohong, Huang Haiting, Lu Xiaoyan, Wu Peifen, Suiyi Zou, Liu Zhenyan

**Affiliations:** 1 Wuchuan People's Hospital, Wuchuan, China; 2 The Fifth People's Hospital of Shunde District, Foshan, China

**Keywords:** preeclampsia, placental tissue selectin (E), angiotensin II and its receptors, oxidized lipids, blood pressure, predictive value, preeklampsija, selektin tkiva placente (E), angiotenzin II i njegovi receptori, oksidovani lipidi, krvni pritisak, prediktivna vrednost

## Abstract

**Background:**

The purpose was to analyze the levels of placental tissue selectins (E), angiotensin II (AngII) and its receptors (ATRs), and oxidized lipids (malondialdehyde (MDA), 8-isoprostane 2a) in patients with preeclampsia (PE). (8-iso-PGF2a)) correlation and clinical significance.

**Methods:**

Select 30 PE pregnant women who were admitted to our hospital from March 2023 to January 2024 as the case group, and select another 30 normal pregnant women who were registered in our hospital during the same period as the health group .The general information of the two groups and placental tissue selectin (E), plasma AngII, ATRs, placental tissue MDA, 8-iso-PGF2a and blood pressure levels (systolic blood pressure (SBP), diastolic blood pressure (DBP)) were compared. Pearson correlation was used to analyze the correlation between the expression of selectin (E), AngII, ATRs, MDA, 8-iso-PGF2a and the levels of SBP and DBP. ROC curves were drawn to analyze the value of placental tissue selectin (E), AngII, ATRs, MDA, and 8-iso-PGF2a individually and jointly in predicting the risk of PE.

**Results:**

The expression of placental tissue selectin (E), AngII, ATRs, MDA, 8-iso-PGF2a and the levels of SBP and DBP in the case group were higher than those in the healthy group (P<0.05). Pearson correlation showed that the expression levels of placental tissue selectin (E), AngII, ATRs, MDA, and 8-iso-PGF2a were positively correlated with SBP and DBP (r>0, P<0.05). The results of drawing the ROC curve showed that the AUCs of placental tissue selectin (E), AngII, ATRs, MDA, and 8-iso-PGF2a expression in predicting the occurrence of PE were 0.854, 0.756, 0.745, 0.885, 0.900, and 0.905 respectively.

**Conclusions:**

Placenta tissue selectin (E), AngII, ATRs, MDA, and 8-iso-PGF2a are highly expressed in pregnant women with PE. The expression of the above indicators is related to maternal blood pressure levels, and their combination can effectively increase predictive value of the risk of PE.

## Abstract (sr)

**Uvod:**

Cilj je bio da se analiziraju nivoi selektina tkiva placente (E), angiotenzina II (AngII) i njegovih receptora (ATR) i oksidisanih lipida (malondialdehid (MDA), 8-izoprostan 2a) kod pacijenata sa preeklampsijom (PE). (8-iso-PGF2a)) korelacija i klinički značaj.

**Metode:**

Odabrano je 30 trudnica sa preeklampsijom (PE) koje su primljene u našu bolnicu od marta 2023. do januara 2024. godine kao grupu slučajeva i izabrano još 30 normalnih trudnica koje su bile registrovane u našoj bolnici u istom periodu kao i zdravstvena grupa. Upoređene su dve grupe i selektin tkiva placente (E), AngII plazme, ATR, MDA tkiva placente, 8-iso-PGF2a i nivoi krvnog pritiska (sistolni krvni pritisak (SBP), dijastolni krvni pritisak (DBP)). Pirsonova korelacija je koriščena za analizu korelacije između ekspresije selektina (E), AngII, ATRs, MDA, 8-iso-PGF2a i nivoa SBP i DBP ROC krive su nacrtane da bi se analizirala vrednost selektina tkiva placente (E), AngII, ATR, MDA i 8-iso-PGF2a pojedinačno i zajedno u predviđanju rizika od PE.

## Introduction

Preeclampsia (PE) is a common complication during pregnancy, with elevated blood pressure as the typical symptom and often causing changes in coagulation function. It is one of the important causes of adverse pregnancy outcomes for mothers and infants and seriously threatens the life and health of mothers and infants [1] [2]. At present, there is no effective clinical treatment for PE other than pregnancy termination. The existing treatment options are mainly to control the progression of the disease and strive to extend the gestational age. Therefore, exploring fast and efficient markers for predicting the occurrence and development of PE is a current research hotspot. Studies have shown that endothelial dysfunction caused by abnormal activation of the renin-angiotensin system (RAS) is closely related to the pathogenesis of PE [3]. Some studies have also pointed out that angiotensin II (AngII) and its receptors (ATRs) are highly expressed in the placental tissue of PE patients, which can cause vasoconstriction and thereby activate placental RAS [4] [5]. Some scholars have pointed out that selectin is a protein that plays an important role in the pathogenesis of eclampsia, and the expression level of endothelial (E) selectin is significantly increased in chronic hypertensive patients with the most severe endothelial dysfunction [6]. Other studies have pointed out that there is also a close regulatory relationship between RAS and selectin (E) [7]. In view of the relationship between AngII, ATRs and selectin (E), RAS and hypertension, it is suggested that they may be used as effective predictors of PE. In recent years, studies have also found that lipid oxidative stress indicators such as malondialdehyde (MDA) and 8-isoprostane 2a (8-iso-PGF2a) in the serum of pregnant women with PE have changed significantly, especially in patients with severe PE [8]. It is suggested that the oxidative stress response mediated by MDA and 8-iso-PGF2a is involved in the pathogenesis of PE. However, there are currently few reports on the correlation between AngII, ATRs, selectin (E), MDA, and 8-iso-PGF2a in placental tissue to predict the risk of PE. Based on this, this study analyzed the expression of AngII, ATRs, selectin (E), MDA, and 8-iso-PGF2a in the placenta tissue of pregnant women with PE, and evaluated the correlation between the above indicators and PE and the value of predicting the risk of PE.

## Materials and methods

### General information

Select 30 PE pregnant women who were admitted to our hospital from March 2023 to January 2024 to be included in the case group, and select another 30 normal pregnant women who were registered in our hospital during the same period to be included in the health group.

### Inclusion criteria for case group

(1) Inclusion criteria. PE meets relevant diagnostic standards; Female aged 18–35; Single birth; Inclusion in the study is at gestational age of 28 weeks or more; Family members have informed consent and are highly cooperative with the research. (2) Exclusion criteria. Those with a history of prenatal hypertension and taking antihypertensive drugs. However, it does not include patients who have been hospitalized for a diagnosis of PE and treated severe hypertension with anti-hypertensive or magnesium sulfate; major fetal structural abnormalities or chromosomal abnormalities confirmed by prenatal diagnosis; diagnosis of gestational type I and type II diabetes, or pregnancy Pregnant women with serum creatinine of 1.2 mg/dL, or systemic lupus erythematosus, or other autoimmune diseases; pregnant women with previous non-pregnancy proteinuria or hypertension; cesarean section due to placental abruption or bleeding complications; Request to withdraw during the research period. This study has received hospital ethics approval (No. 20190042).

### Method

Placental tissue is collected after the woman gives birth. Use a scalpel to cut out 2 pieces of placenta tissue of 1 cm×1 cm×1 cm size. After washing with physiological saline, absorb the water with filter paper, then put one part into 4% formaldehyde solution for fixation and paraffin embedding. For immunohistochemical staining; place one portion into an EP tube wrapped in tin foil, and then store it in a -80°C refrigerator for detection of oxidized lipid substances. Label before storing.

### Plasma sample collection

Maternal plasma was collected at enrollment. Collect 5 mL of peripheral venous blood through a blood collection device, let it stand at room temperature for 30 min, and then centrifuge at 4000 rpm for 5 min. Collect the supernatant into a sterile EP tube, mark it, and store it in a -80°C refrigerator.

### Placental tissue selectin (E), AngII, ATRs, MDA, and 8-iso-PGF2a expression

Remove the placental tissue from the refrigerator and thaw at room temperature. (1) Selectin (E) Immunohistochemical SP method (Beijing Zhong shan Biotechnology Co., Ltd.) was used, and the placenta tissue was subjected to paraffin sectioning and immunohistochemical staining in strict accordance with the instructions. The Powersite microscopic image acquisition and analysis system (Shanghai Shanfu Scientific Instrument Co., Ltd.) was used to calculate the average optical density of selectin (E) in the syncytiotrophoblast of placental villi in each high-power field, and the average optical density of each placenta specimen was obtained. This represents the selectin (E) expression level in placental tissue. (2) Competitive enzyme-linked immunosorbent assay technology (Kit: Shanghai Shenggong Biotechnology Services Co., Ltd., D711342) was used to measure AngII and ATRs (mRNA) levels in strict accordance with the instructions. (3) After the samples are collected, the whole sample is sent for testing, and the expression levels of MDA and 8-iso-PGF2a are detected with the help of the metabolomics detection platform of the Basic Medical Experimental Department of Southern Medical University.

### Coagulation indicators

The plasma was taken out from the refrigerator, thawed at room temperature, Measure the levels of thromboplastin time (APTT), platelet count (PLT), and fibrinogen (FIB) using the Hizen Mikon CS5100 fully automated coagulation analyzer from Japan.

### PE treatment

Dissolve 2.5–5.0 g of magnesium sulfate (national drug approval number H20033861, Hebei Tiancheng Pharmaceutical Co., Ltd., specification: 10 mL: 2.5 g) in 5% and 20 mL glucose injection for intravenous infusion of shock therapy (slowly completed within 15–20 minutes), then dissolve 15 g of magnesium sulfate in 5% and 500 mL glucose injection for intravenous infusion (drip rate 1.0–2.0 g/h) for maintenance therapy; Oral Labelol Hydrochloride Tablets (Zhengzhou Kaili Pharmaceutical Co., Ltd., National Medical Standard H41024906, Specifi cation: 50 mg), 100 mg/time, 3 times/day. If the effect is not good, it can be adjusted to 200 mg/time, 3 times/day; Oral Aspirin Enteric coated Tablets (National Medical Standard H43021814, Shutaishen (Beijing) Biopharmaceutical Co., Ltd., Specification: 50 mg), 100 mg/time, once a day. During treatment, pay close attention to changes in the patient’s blood pressure, urine output, and routine hematuria, and pay attention to the monitoring of fetal movement and fetal heart rate. All were treated until delivery.

### General information

General information such as age, body mass index, smoking history, parity, blood pressure, 24-hour urine protein quantification, proteinuria protein value, gestational age at delivery, fetal weight, Cesarean section ratio, caesarean section ratio, neonatal Apgar score, etc. were recorded in the case group and the healthy group.

### Statistical analysis

Statistic Package for Social Science (SPSS) 23.0 statistical analysis (IBM, Armonk, NY, USA) software was used, and the measurement data were described as mean ± standard deviation (x̄±S) and tested with *t*. Bivariate Pearson linear correlation was used to test the correlation between placental tissue selectin (E), AngII, ATRs, MDA, 8-iso-PGF2a and blood pressure levels; Draw a ROC curve to analyze the value of placental tissue selectin (E), AngII, ATRs, MDA, and 8-iso-PGF2a individually and jointly in predicting the risk of PE. AUC value > 0.9 indicates high predictive performance, and 0.71–0.9 indicates certain prediction. Perfor mance, 0.5–0.7 indicates low prediction performance, < 0.5 indicates no prediction, and P < 0.05 indicates that the difference is statistically significant.

## Results

### Comparison of relevant data between healthy group and case group

**Figure 1:**
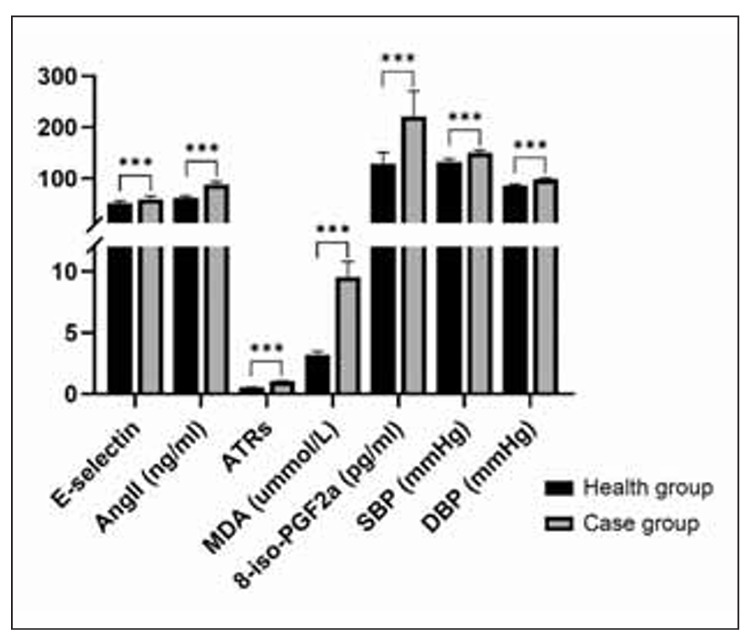
Comparison of the expression levels of selectin (E), AngII, ATRs, MDA, 8-iso-PGF2a and the levels of SBP and DBP in the two groups (***P<0.001).

Comparison of age, pre-pregnancy body mass index, smoking history, parity, blood pressure, 24-hour urine protein quantification, proteinuria protein value, gestational age at delivery, fetal weight, caesarean section proportion, neonatal Apgar score, APTT, FIB, and PLT between healthy group and case group , the difference is not statistically significant (P>0.05); The expression of placental tissue selectin (E), AngII, ATRs, MDA, 8-iso-PGF2a and the levels of SBP, 24-hour urine protein quantification and DBP in the case group were higher than those in the healthy group (P<0.05). See Table 1 and Figure 1.

**Table 1:** Comparison of relevant data between healthy group and case group (x̄±S).

index	Healthy group<br>(n=30)	Case group<br>(n=30)	Statistical values	*P value*
Selectin (e) (ng/mL)	50.96±5.10	58.80±6.15	5.375	0.000
AngII (ng/mL)	60.80±5.13	87.55±6.20	18.270	0.000
ATRs (ng/mL)	60.80±5.13	87.55±6.20	18.270	0.000
MDA (μmmol/L)	3.24±0.28	9.56±1.24	27.231	0.000
8-iso-PGF2a (pg/mL)	128.32±22.25	221.34±50.24	9.273	0.000
SBP (mmHg)	132.57±5.37	149.63±5.20	12.498	0.000
DBP (mmHg)	86.10±2.06	97.63±3.01	17.322	0.000
Age (years)	25.00±2.21	25.03±2.17	0.059	0.953
24-hour urine protein<br>quantification (mg)	200.63±25.39	400.41±25.52	30.397	0.000
Gestational age at delivery (weeks)	35.90±1.27	35.83±1.26	0.204	0.839
Fetal weight (kg)	3.03±0.35	3.05±0.33	0.228	0.821
Apgar score of newborn (score)	8.07±0.52	8.00±0.64	0.441	0.661
FIB (g/L)	3.49±0.26	3.52±0.29	0.496	0.622
APTT (s)	38.76±3.23	37.59±3.30	1.388	0.176
PLT (×10^9^/L)	250.73±20.73	259.87±22.68	1.628	0.109
Precursor mass index >24 kg/m^2^	10 (33.33)	14 (46.67)	1.111	0.292
24 kg/m^2^	20 (66.67)	16 (53.33)		
Smoking history have	0 (0.00)	2 (6.67)	0.517	0.472
without	30 (100.00)	28 (93.33)		
Parity primiparity	16 (53.33)	18 (60.00)	0.272	0.602
multiparity	14 (46.67)			
	12 (40.00)			
Cesarean section be	18 (60.00)	20 (66.67)	0.287	0.592
no	12 (40.00)	10 (33.33)		

### Correlation analysis between placental tissue selectin (E), AngII, ATRs, MDA, 8-iso-PGF2a expression and SBP and DBP levels

**Figure 2:**
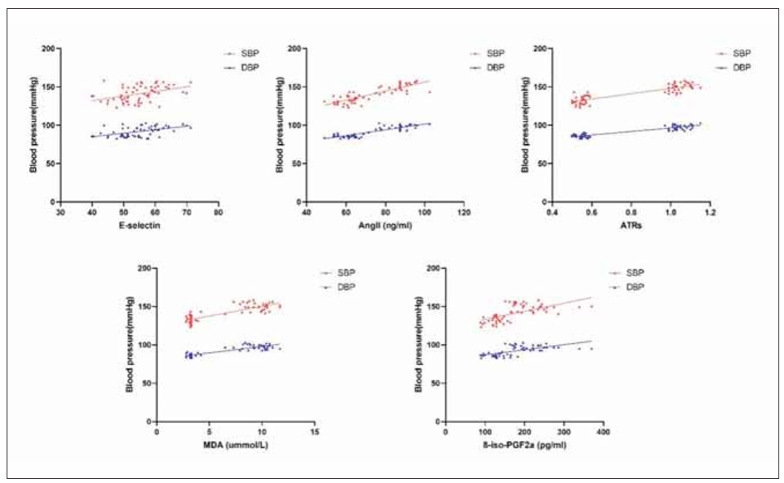
Correlation analysis between placental tissue selectin (E), AngII, ATRs, MDA, 8-iso-PGF2a expression and SBP and DBP levels.

Pearson correlation showed that the expression levels of placental tissue selectin (E), AngII, ATRs, MDA, and 8-iso-PGF2a were positively correlated with SBP and DBP (r>0, P<0.05). See Table 2 and Figure 2.

**Table 2:** Correlation analysis between placental tissue selectin (E), AngII, ATRs, MDA, 8-iso-PGF2a expression and SBP and DBP levels.

Index	Coefficient	Selectin<br>(E)	AngII	ATRs	MDA	8-iso-<br>PGF2a
SBP	*r*	0.221	0.444	0.562	0.453	0.235
	* P *	0.003	0.000	0.000	0.000	0.001
DBP	* r *	0.223	0.447	0.621	0.449	0.232
	* P *	0.003	0.000	0.000	0.000	0.002

### Analysis of the value of placental tissue selectin (E), AngII, ATRs, MDA, and 8-iso-PGF2a expression levels individually and jointly in predicting the risk of PE

**Figure 3:**
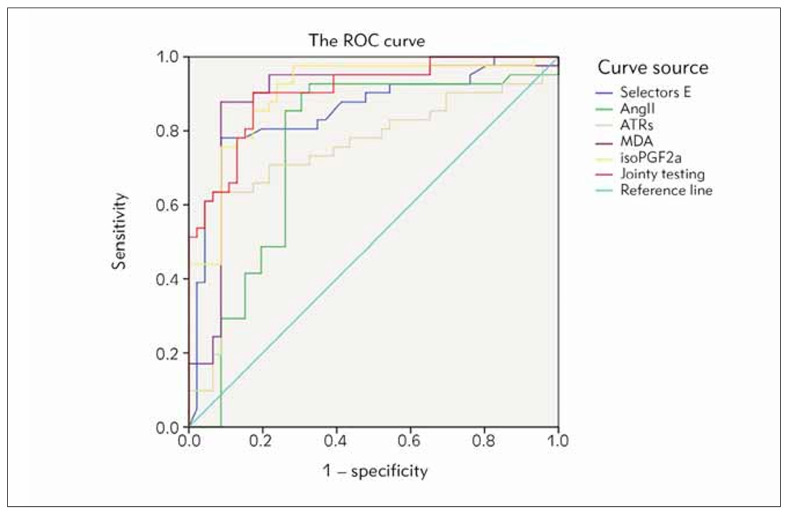
ROC chart for predicting the risk of PE occurrence.

Taking the status of PE occurrence (0 means occurred, 1 means not occurring) as the state variable, and the expression of placental tissue selectin (E), AngII, ATRs, MDA, and 8-iso-PGF2a as the test variables, the ROC curve results found that, the AUCs of placental tissue selectin (E), AngII, ATRs, MDA, and 8-iso-PGF2a expression in predicting the risk of PE were 0.854, 0.756, 0.745, 0.885, 0.900, and 0.905. See Table 3 and Figure 3.

**Table 3:** Analysis of the value of placental tissue selectin (E), AngII, ATRs, MDA, and 8-iso-PGF2a expression alone and jointly in predicting the risk of PE.

Target	Item	Optimal cutoff<br>value	AUC	Standard error	P	95%CI
PE risk	selectin (E) (ng/mL)	85.622	0.854	0.043	0.000	0.769–0.938
	AngII ng/mL	1.050	0.756	0.057	0.000	0.643–0.865
	ATRs ng/mL	9.225	0.745	0.056	0.000	0.635–0.855
	MDA μmmol/L	221.430	0.885	0.042	0.000	0.803–0.967
	8-iso-PGF2a pg/mL	150.455	0.900	0.034	0.000	0.836–0.970
	Joint	96.905	0.905	0.032	0.000	0.842–0.967

## Discussion

PE is a pregnancy-specific syndrome, with hypertension and proteinuria occurring after 20 weeks of pregnancy as the main pathological features. The global incidence rate is 2–8% [9]. PE not only increases the risk of cardiovascular, metabolic, and renal diseases for mothers and their children, but is also the leading cause of maternal perinatal death. Studies have shown that placental abnormalities, oxidative stress, extensive activation of leukocytes, and endothelial dysfunction caused by abnormal activation of RAS are all closely related to the onset of PE [10] [11] [12]. Some studies have pointed out that selectin (E) is a member of the selectin family, which can mediate abnormal activation of leukocytes through endothelial cells [13]. Some studies have pointed out that RAS is the main system for regulating blood pressure balance in the body. In addition to the classic RAS, there is a local independent RAS in maternal placental tissue. The expression of AngII and ATRsmRNA in placental tissue can regulate placental blood circulation, which is related to The occurrence and development of PE are closely related [14] [15]. Some studies have also pointed out that the oxidative stress reaction in placental tissue mediated by lipid oxidative stress indicators such as MDA and 8-iso-PGF2a is an independent risk factor for PE [8] [16]. The above studies suggest that selectin (E), AngII, ATRsmRNA, MDA, and 8-iso-PGF2a may become effective indicators for predicting the occurrence and development of PE. The results of this study showed that the expression of placental tissue selectin (E), AngII, ATRs, MDA, 8-iso-PGF2a and the levels of SBP and DBP in the case group were higher than those in the healthy group; Pearson correlation showed that the expression levels of placental tissue selectin (E), AngII, ATRs, MDA, and 8-iso-PGF2a were positively correlated with SBP and DBP. It is suggested that selectin (E), AngII, ATRs, MDA, and 8-iso-PGF2a are highly expressed in the placenta tissue of pregnant women with PE and have certain value in predicting PE. Analysis of the possible reasons is: (1) Under pathological conditions, damaged endothelial cells will overexpress selectin (E), prompting a large number of white blood cells to be activated, aggregate and cross the placental endothelial cells, exacerbating the degree of endothelial cell damage; at the same time, activated white blood cells It will continue to migrate and produce a large amount of biologically active products, prompting the adhesion and secretion of selectin (E), further aggravating the damage to the endothelial function of placental tissue, and ultimately causing PE. (2) AngII and AT1R are important components of RAS. Increased levels of both can cause corresponding physiological effects, including: 1) Promote the continuous contraction of arterioles and veins throughout the body, thereby causing the blood pressure to continuously increase and the amount of blood returned to the heart to increase; 2) Increase the release of fibrous transmitter from sympathetic vasoconstrictors and promote central nervousness of sympathetic vasoconstrictors; 3) Stimulates the adrenal gland to synthesize and release aldosterone, continuously constricts blood vessels, thereby destroying placental RAS and increasing the risk of PE [4] [14] [17]. (3) High levels of MDA and 8-iso-PGF2a in pregnant women are involved in abnormal pathological changes in the placenta and activate inflammatory responses, aggravating the body’s oxidative damage, directly damaging the body’s endothelial cells, reducing prostacyclin synthesis, and thromboxane. The synthesis increases and the ratio of prostacyclin/thromboxane decreases, ultimately causing arteriovenous vasoconstriction and increasing the sensitivity of blood vessels to RAS, leading to a series of clinical symptoms and pathology of PE such as placental tissue damage, increased blood viscosity, hypertension, and proteinuria. Physiological changes [18]. Therefore, overexpression of placental tissue selectin (E), AngII, ATRs, MDA, and 8-iso-PGF2a can continuously aggravate placental tissue damage through excessive activation of leukocytes, abnormal activation of placental tissue RAS, abnormal pathological changes in the placenta, and aggravation of oxidative stress response. The degree of tissue endothelial function damage, the degree of endothelial dysfunction in pregnant women is more severe, and the risk of PE is higher. This study further drew the ROC curve and found that the AUCs of placental tissue selectin (E), AngII, ATRs, MDA, and 8-iso-PGF2a expression in predicting the risk of PE were 0.854, 0.756, 0.745, 0.885, 0.900, and 0.905. The results showed that the placental tissue selectin (e) , AngII, ATRs, MDA, 8-iso-PGF2a were the effective predictors of the risk of PE, but the combination of them was the best. The reason is that many factors, such as drug factors, maternal hormone level, external environment and so on, can cause the above-mentioned indexes to be abnormal, and then affect the prediction efficiency, the above-mentioned indexes can complement each other, reduce the bad influence of single index abnormal expression on the overall prediction accuracy, and effectively improve the effectiveness of predicting the risk of PE. Although this study analyzed the correlation and clinical significance of placental tissue selectin (e) , Angiotensino gen II and its receptors, and levels of oxidized lipids in patients with preeclampsia, there are still some limitations in this study, the study included: (1) the study sample size was small, the study time was short, and the study object source was limited by region; (2) the study object took the 18–35 year-old pregnant women as the study object, did not include the > 35 year-old pregnant women; (3) the basic data of pregnant and lying-in women were not further screened, and the above factors may influence the results of the study. Therefore, in future studies, it is necessary to increase the sample size, extend the study period, break the age limit of the sample size, expand the source of the sample size, and further conduct multicenter Randomized controlled trial, to confirm the results of this study.

## Dodatak

### Funding

This work was supported by the Foshan Science and Technology Bureau 2022 research Project, project number: 2220001004868.

### Conflict of interest statement

All the authors declare that they have no conflict of interest in this work.

## References

[1] Roberts J M, Rich-Edwards J W, McElrath T F, Garmire L, Myatt L (2021). Subtypes of preeclampsia: Recognition and determining clinical usefulness. Hypertension.

[2] Ives C W, Sinkey R, Rajapreyar I, Tita A, Oparil S (2020). Preeclampsia-pathophysiology and clinical presentations: JACC state-of-the-art review. J Am Coll Cardiol.

[3] Vargas V R, Varela M J, Fajardo B E (2022). Renin-angiotensin system: Basic and clinical aspects: A general perspective. Endocrinol Diab Nutr.

[4] Biwer L A, Lu Q, Ibarrola J, Stepanian A, Man J J, Carvajal B V, et al (2023). Smooth muscle mineralocorticoid receptor promotes hypertension after preeclampsia. Circ Res.

[5] Civieri G, Iop L, Tona F (2022). Antibodies against angiotensin II type 1 and endothelin 1 type A receptors in cardiovascular pathologies. Int J Mol Sci.

[6] Han Q, Zheng S, Chen R, Zhang H, Yan J (2022). A new model for the predicting the risk of preeclampsia in twin pregnancy. Front Physiol.

[7] Marín R, Chiarello D I, Abad C, Rojas D, Toledo F, Sobrevia L (2020). Oxidative stress and mitochondrial dysfunction in early-onset and late-onset preeclampsia. Bba-Mol Basis Dis.

[8] Cabunac P, Karadžov O N, Ardalić D, Banjac G, Ivanišević J, Janać J, et al (2021). Unraveling the role of oxidative stress and lipid status parameters in the onset of preeclampsia. Hypertens Pregnancy.

[9] Wang Y, Li B, Zhao Y (2022). Inflammation in preeclampsia: Genetic biomarkers, mechanisms, and therapeutic strategies. Front Immunol.

[10] Rana S, Lemoine E, Granger J P, Karumanchi S A (2019). Preeclampsia: Pathophysiology, challenges, and perspectives. Circ Res.

[11] Tomimatsu T, Mimura K, Matsuzaki S, Endo M, Kumasawa K, Kimura T (2019). Preeclampsia: Maternal systemic vascular disorder caused by generalized endothelial dysfunction due to placental antiangiogenic factors. Int J Mol Sci.

[12] Jena M K, Sharma N R, Petitt M, Maulik D, Nayak N R (2020). Pathogenesis of preeclampsia and therapeutic approaches targeting the placenta. Biomolecules.

[13] Skrzypczyk P, Ozimek A, Ofiara A, Szyszka M, Sołtyski J, Stelmaszczyk-Emmel A, et al (2019). Markers of endothelial injury and subclinical inflammation in children and adolescents with primary hypertension. Cent Eur J Immunol.

[14] Saez T, Spaans F, Kirschenman R, Sawamura T, Davidge S T (2020). High-cholesterol diet during pregnancy induces maternal vascular dysfunction in mice: potential role for oxidized LDL-induced LOX-1 and AT1 receptor activation. Clin Sci.

[15] Yu Y, Zhang L, Xu G, Wu Z, Li Q, Gu Y, et al (2018). Angiotensin II type I receptor agonistic autoantibody induces podocyte injury via activation of the TRPC6-calcium/calcineurin pathway in pre-eclampsia. Kidney Blood Press R.

[16] Mentese A, Guven S, Demir S, Sumer A, Yaman S O, Alver A, et al (2018). Circulating parameters of oxidative stress and hypoxia in normal pregnancy and HELLP syndrome. Adv Clin Exp Med.

[17] Mazloomi S, Alimohammadi S, Khodadadi I, Ghiasvand T, Shafiee G (2020). Evaluation of methylenetetrahydrofolate reductase (MTHFR) activity and the levels of homocysteine and malondialdehyde (MDA) in the serum of women with preeclampsia. Clin Exp Hypertens.

[18] Chiarello D I, Abad C, Rojas D, Toledo F, Vázquez C M, Mate A, et al (2020). Oxidative stress: Normal pregnancy versus preeclampsia. Bba-Mol Basis Dis.

